# Management and outcomes of cataract surgery in uveitic eyes with chronic hypotony: a two-case series

**DOI:** 10.1186/s12348-025-00554-x

**Published:** 2025-12-29

**Authors:** Fawzia Alhaimi, Walaa Bakhamees

**Affiliations:** 1 Uveitis Division, King Khalid Eye Specialist Hospital, Riyadh, Saudi Arabia; 2 Jeddah University, Jeddah, Saudi Arabia

**Keywords:** Uveitis, Chronic hypotony, Cataract surgery, Immunosuppression, Aphakia

## Abstract

**Background:**

Cataract surgery in uveitic eyes with persistent hypotony is considered high-risk and is often deferred. This report describes the perioperative management and visual outcomes in such complex cases.

**Objective:**

To evaluate the effectiveness of a tailored surgical and immunosuppressive approach for patients with uveitis, cataracts, and chronic hypotony (IOP ≤ 5 mmHg).

**Methods:**

A retrospective review of two cases: a 21-year-old female with chronic intermediate non-granulomatous uveitis and a 17-year-old male with Vogt-Koyanagi-Harada (VKH) disease. Both developed bilateral cataracts and persistent hypotony. A multi-step protocol was implemented, involving aggressive immunosuppression (azathioprine/methotrexate plus adalimumab) and an average of two periocular triamcinolone acetonide (40 mg/mL) injections per eye to elevate IOP to a safe surgical threshold (≥ 8 mmHg). After achieving ≥ 3 months of quiescent inflammation and normalized IOP, patients underwent lens aspiration with intravitreal triamcinolone injection and were intentionally left aphakic.

**Results:**

Preoperative IOP was successfully elevated to a mean of 10 mmHg. One eye experienced an intraoperative complication (dropped nucleus) requiring pars plana vitrectomy; this eye later developed corneal decompensation necessitating penetrating keratoplasty. Postoperative inflammation resolved within one week in all eyes. On long-term follow-up (mean 24 months), inflammation remained controlled on maintenance immunosuppression. At the two-year follow-up, best-corrected visual acuity was 20/30 in three eyes and 20/50 in the eye that required additional surgeries. IOP was maintained at ≥ 8 mmHg in all eyes.

**Conclusion:**

A meticulously planned, multi-modal approach—involving aggressive control of inflammation, targeted reversal of hypotony with periocular steroids, and strategic surgical timing with intentional aphakia—can lead to successful anatomical and visual outcomes in high-risk uveitic patients with cataracts and persistent hypotony.

## Introduction

Cataract formation is a frequent and sight-threatening complication in patients with uveitis, with a reported incidence ranging from 8.5% to 35% [1]. This process is driven by chronic intraocular inflammation and the long-term use of corticosteroid therapy [1]. Performing cataract surgery in these patients is notoriously complex, as pre-existing ocular comorbidities often compromise surgical outcomes [1, 2].

A particularly formidable challenge is the co-existence of ocular hypotony, defined as a persistent intraocular pressure (IOP) below 6.5 mmHg. Hypotony occurs in 1.2% to 10% of uveitis patients and arises from diverse pathophysiological mechanisms, including acute ciliary body shutdown, enhanced uveoscleral outflow, and chronic, irreversible damage such as ciliary body atrophy or mechanical traction from cyclitic membranes [3, 4]. Such damage manifests as ciliary body atrophy, a condition notably observed in Vogt-Koyanagi-Harada (VKH) syndrome, where uncontrolled granulomatous cyclitis leads to ischemic and fibrotic destruction of aqueous-producing tissue [5–8]. An alternative mechanism is mechanical traction from cyclitic membranes, which detaches and distorts the ciliary body, leading to functional failure [9–11].

The surgical management of cataracts in uveitic eyes with concomitant hypotony thus presents a unique set of challenges. Meticulous preoperative control of inflammation for a minimum of three months is considered crucial for surgical success [1, 2]. Intraoperatively, surgeons must navigate constricted pupils, posterior synechiae, and zonular weakness [1, 2]. Postoperatively, these eyes remain at elevated risk for complications such as cystoid macular edema (CME), posterior capsule opacification (PCO), and recurrent inflammation [1, 2, 12].

While the outcomes of cataract surgery in quiescent uveitis have been extensively studied [1, 2, 12], a significant gap remains in the literature regarding surgical results in eyes with pre-existing hypotony [3, 4]. This case series aims to address this gap by describing a structured perioperative protocol and its visual outcomes, highlighting key strategic considerations for optimizing visual rehabilitation in this high-risk surgical population.

## Case series

**Fig. 1:**
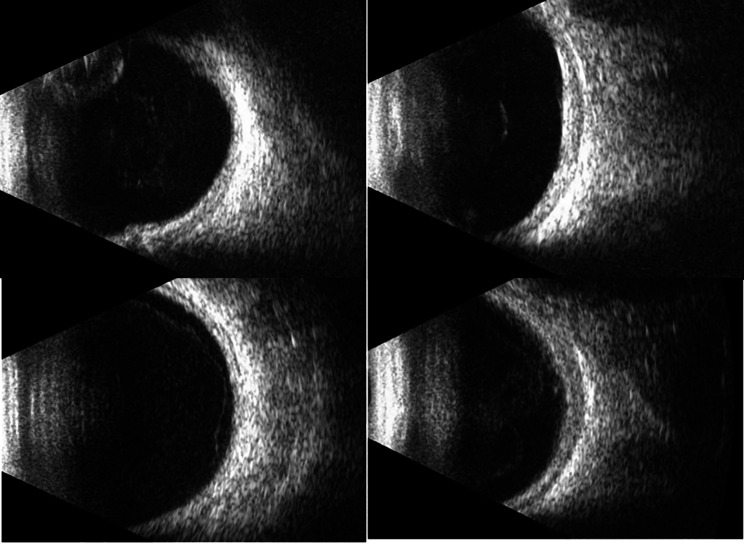
Shows preoperative B-scans for two patients, with the top row (**A**-left, **B**-right) for Pt 1’s eyes and the bottom row (**A**-left, **B**-right) for Pt 2’s eyes

**Fig. 2:**
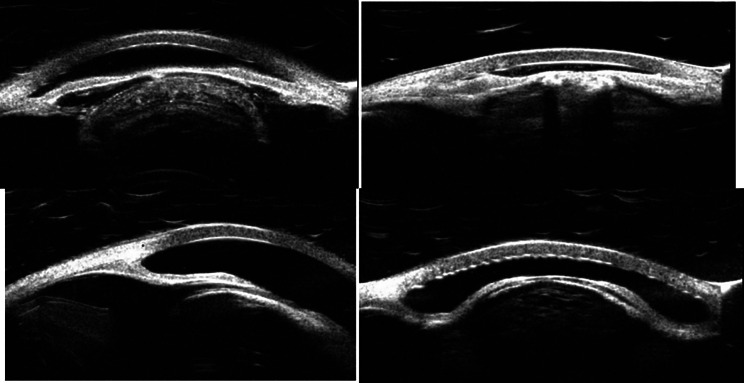
Shows preoperative UBM images for two patients, with the top row (**A**-left, **B**-right) for Pt 1’s eyes and the bottom row (**A**-left, **B**-right) for Pt 2’s eyes

This report details two patients presenting with a triad of bilateral cataracts, persistent hypotony (IOP ≤ 5 mmHg), and quiet anterior segments. Diagnostic B-scan ultrasonography revealed a flat, attached retina with diffuse ocular wall thickening, consistent with the choroidal thickening seen in chronic hypotony (Fig. 1). Ultrasound biomicroscopy (UBM) demonstrated flat, apposed ciliary bodies without detachment or cyclitic membranes; however, the bodies appeared functionally impaired, consistent with inflammatory shutdown (Fig. 2). The study was conducted with appropriate ethical approval and informed consent.

### Case 1

**Fig. 3:**
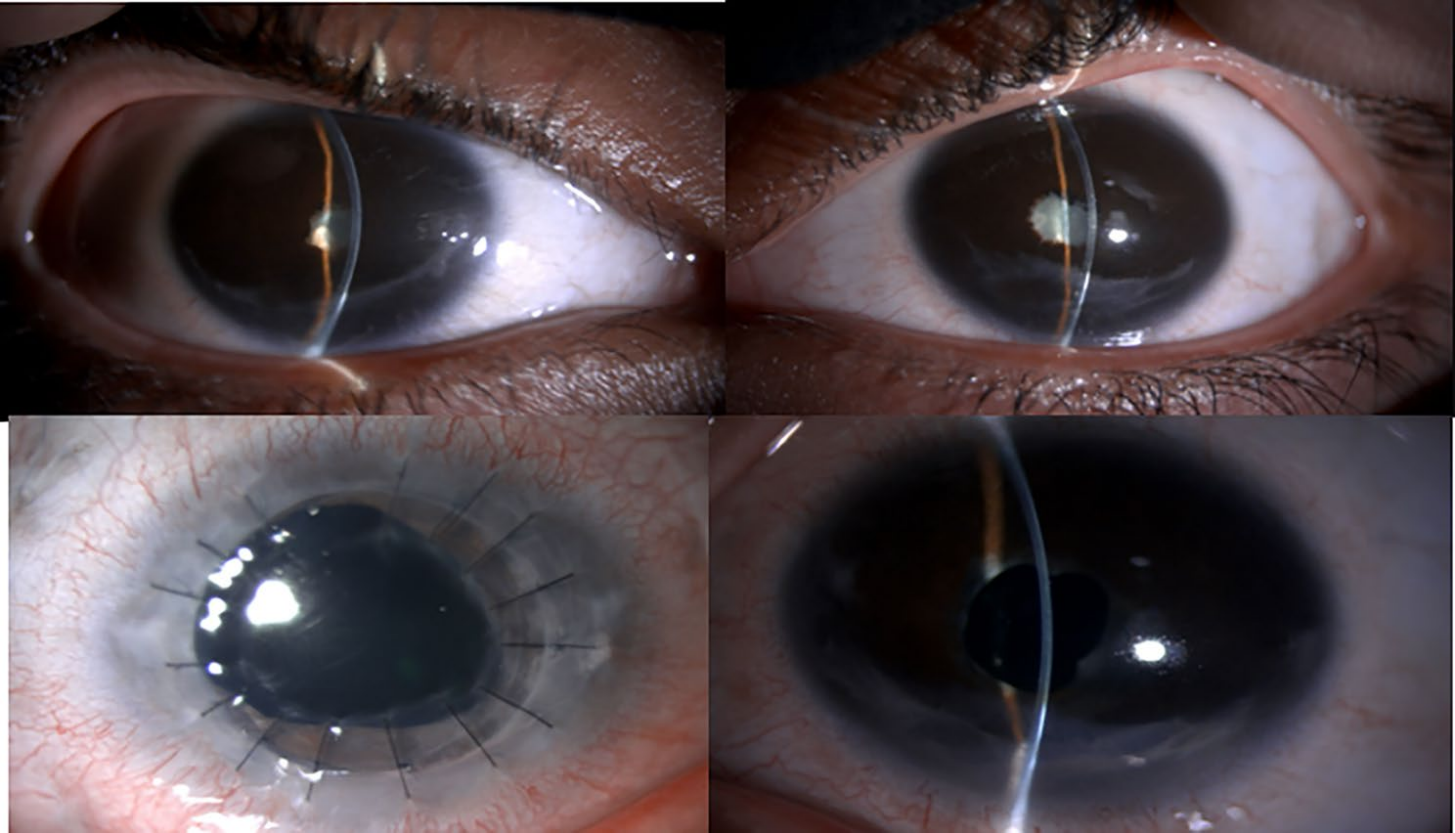
For patient 1. Top Row: Pre-operative views (**A**-right eye, **B**-left eye); Bottom Row: Post-operative views (**C**-right eye, **D**-left eye)

**Fig. 4:**
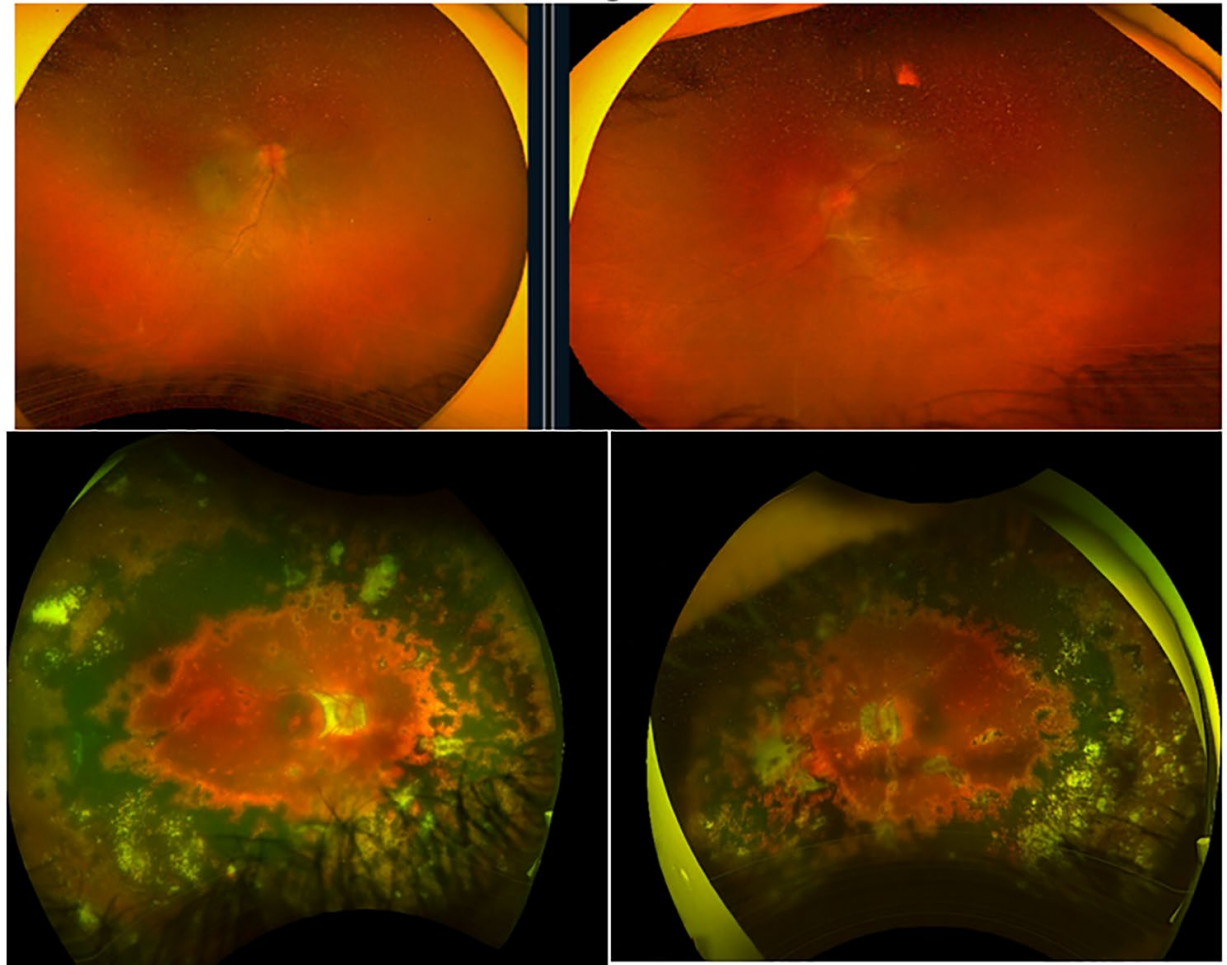
Shows fundus photos for two patients: Pt 1 (top row) has images of the right (**A** and left (**B**) eye, while Pt 2 (bottom row) has images of the right (**A**) and left (**B**) eye

A 21-year-old female was initially diagnosed with bilateral anterior non-granulomatous uveitis and treated with topical prednisolone for four years. She developed secondary chronic hypotony and bilateral cataracts, which were initially deemed inoperable. At presentation, her visual acuity was hand motion bilaterally, with IOP consistently measuring 5 mmHg in both eyes. Examination revealed clear corneas, very shallow anterior chambers, 360-degree peripheral anterior synechiae, posterior synechiae, and dense cataracts (worse in the right eye). The right eye had a similar short axial length (21.2 mm). B-scan ultrasonography confirmed diffuse, significant scleral thickening in both eyes, consistent with the chronic inflammatory state (Figs. 3 and 4).

Treatment was intensified with oral azathioprine (50 mg twice daily), adalimumab (40 mg every two weeks), hourly topical prednisolone acetate 1%, and atropine three times daily. Both eyes received multiple periocular triamcinolone injections (40 mg/mL), averaging two injections per eye at 4–6 week intervals. This regimen successfully elevated the IOP to 11 mmHg in both eyes.

Biometry for the left eye revealed an axial length of 21.0 mm, with standard formulas calculating an IOL power of approximately 32.0 D. Given the context of significant ocular hypotony and short axial length, the risk of a substantial refractive surprise was deemed high. The decision was made to proceed with lens aspiration and leave the patient aphakic. As the surgical plan for aphakia was thus firmly established for both eyes, formal axial length measurement of the fellow eye was not performed, as it would not have altered our management.

Under coverage of high-dose systemic corticosteroids (1 g IV methylprednisolone daily for three days preoperatively, then switched to oral prednisolone 1 mg/kg), the left eye underwent an uncomplicated lens aspiration, combined with intravitreal triamcinolone (4 mg in 0.1 mL), sub-Tenon’s triamcinolone (40 mg), and subconjunctival dexamethasone (2 mg/0.5 ml) injections. Postoperative corrected visual acuity improved to 20/125, with an IOP of 13 mmHg.

One week later, the same procedure was attempted on the right eye. The nucleus was stony hard and firmly adhered to the iris. During manipulation, the nucleus was dislocated into the vitreous cavity. A pars plana vitrectomy (PPV) was performed one week later to remove the dropped nucleus. Fundus examination during vitrectomy revealed peripheral snowballs, confirming the presence of intermediate uveitis; intravitreal triamcinolone was administered. Postoperative visual acuity was 20/400 due to corneal decompensation. The patient subsequently underwent penetrating keratoplasty, achieving an uncorrected visual acuity of 20/100. In the left eye, vision later decreased due to PCO; YAG laser capsulotomy was successfully performed.

At the two-year follow-up, best-corrected visual acuity was 20/50 in the right eye and 20/25 in the left. IOP was 10 mmHg in the right eye and 8 mmHg in the left.

### Case 2

A 16-year-old male with Vogt-Koyanagi-Harada (VKH) disease in the convalescent phase presented with bilateral cataracts and chronic hypotony. His treatment included weekly methotrexate injections (20 mg) and multiple intravitreal bevacizumab injections for a peripapillary choroidal neovascular membrane. Visual acuity was hand motion in both eyes, with a mean baseline IOP of 4–5 mmHg.

Examination revealed early band keratopathy, very shallow and quiet anterior chambers, 360-degree peripheral anterior synechiae, posterior synechiae, and dense cataracts (worse in the right eye). B-scan ultrasonography revealed diffuse ocular wall thickening, and UBM demonstrated flat ciliary bodies, consistent with the findings in Case 1 (Figs. 1 and 2). Formal biometry was not performed as the strategic decision for bilateral aphakia had been established.

Treatment was intensified with adalimumab (40 mg every two weeks) and hourly topical prednisolone acetate 1%. Both eyes received multiple periocular triamcinolone injections (40 mg at least 4 weeks apart), which increased the IOP to 9 mmHg in both eyes.

Under coverage of high-dose systemic corticosteroids (1 g IV methylprednisolone daily for three days, then switched to oral prednisolone 1 mg/kg), the patient underwent uneventful bilateral lens aspiration combined with intravitreal triamcinolone (4 mg in 0.1 mL), sub-Tenon’s triamcinolone (40 mg), and subconjunctival dexamethasone (2 mg/0.5 ml). The procedures were performed one week apart.

Postoperative inflammation resolved within one week without uveitis exacerbation. At the two-year follow-up, corrected visual acuity was 20/30 in both eyes, and IOP was maintained at 10 mmHg in the right eye and 11 mmHg in the left.

### Postoperative immunosuppressive regimen

The high-dose oral prednisolone was tapered to a maintenance dose of 5-7.5 mg daily by three months. Topical steroids were tapered to twice daily by three months. No additional periocular or intravitreal steroid injections were required. The systemic immunomodulatory therapy (azathioprine, methotrexate, and adalimumab) was continued unabated to ensure sustained disease control and graft stability. The long-term therapeutic strategy includes the future gradual tapering of these immunomodulatory agents, contingent upon clinical stability and the absence of rejection or disease flare.

## Discussion

This report demonstrates that successful visual and IOP outcomes are achievable in the surgical management of uveitic cataracts, even when complicated by severe, persistent hypotony. The significant IOP response to intensive therapy (a near-doubling from baseline) indicates that the pathophysiology was a profound, but potentially reversible, inflammation-induced ciliary body shutdown rather than end-stage fibrotic atrophy. This challenges the conventional view of such hypotony as an absolute contraindication to surgery.

This understanding mandates an aggressive, multi-modal approach to perioperative immunosuppression. Our protocol, which included high-dose local and systemic corticosteroids, conventional immunosuppressants, and biologic agents, was designed to achieve profound inflammatory quiescence for a minimum of three months preoperatively [1, 2, 13]. We further harnessed the ocular hypertensive effect of periocular triamcinolone to therapeutically counteract the hypotony, successfully elevating IOP to a safe surgical range.

The critical importance of this approach is underscored by the significant risk of irreversible ciliary body injury in chronic hypotony [4]. This risk is particularly pertinent in conditions like VKH disease, which is associated with poorer outcomes [3]. Our intensive regimen was pivotal in mitigating these risks, as evidenced by the rapid resolution of postoperative inflammation and the absence of CME—a frequent complication in uveitic cataract surgery [14, 15].

The surgical strategy must also be adapted to these complex anatomical conditions. The decision for aphakic rehabilitation, while non-standard, was a strategic choice justified by multiple factors. Beyond the refractive uncertainty in hypotonus, short eyes, the complete removal of the lens capsule eliminates a potential scaffold for the formation of sight-threatening cyclitic membranes—a significant risk in these inflamed eyes [3, 16]. This approach prioritizes long-term anatomical stability and the prevention of phthisis over immediate refractive correction. This careful, patient-tailored approach allowed for significant visual improvement (20/30 or better in three of four operated eyes), a notable achievement given that uveitic cataracts consistently have worse outcomes than age-related cataracts [17].

Postoperative management requires a careful, prolonged taper of corticosteroids under the cover of sustained immunomodulation, as detailed in our cases. These patients need lifelong monitoring for inflammation recurrence and complications like glaucoma. Regarding future implantation, secondary intraocular lens (IOL) surgery can be considered once the eye has demonstrated long-term stability off high-dose steroids, with normalized IOP for a period of 12–24 months. The options include a scleral-fixated or an anterior chamber IOL, with the former often preferred in these complex eyes to avoid angle compromise.

## Conclusion

In conclusion, our report demonstrates that successful visual rehabilitation (20/30 or better in three of four operated eyes) is attainable in uveitic cataracts complicated by severe, persistent hypotony. This success is predicated on a management protocol that directly targets the underlying pathophysiology of inflammatory ciliary body shutdown. Through aggressive, sustained immunosuppression and surgical strategies adapted to anatomical risk, including deliberate aphakia, we have reframed chronic hypotony from an absolute surgical contraindication into a critical factor mandating intensive pre-operative optimization. This paradigm shift, centering on controlling the inflammatory drive, provides a viable framework for managing these most challenging cases.

## Data Availability

No datasets were generated or analysed during the current study.
